# Microbial composition in *Hyalomma anatolicum* collected from livestock in the United Arab Emirates using next-generation sequencing

**DOI:** 10.1186/s13071-021-05144-z

**Published:** 2022-01-20

**Authors:** Nighat Perveen, Sabir Bin Muzaffar, Ranjit Vijayan, Mohammad Ali Al-Deeb

**Affiliations:** Biology Department, College of Science, United Arab Emirates University, P.O. Box 15551, Al-Ain, United Arab Emirates

**Keywords:** *Hyalomma anatolicum*, *16S* rRNA gene, Microbial communities, Pathogens, Livestock

## Abstract

**Background:**

*Hyalomma anatolicum* is a widely distributed tick species that acts as a vector transmitting tick-borne pathogens (TBPs) in livestock. Such pathogens affect the health of livestock and consequently reduce their productivity. Knowledge about the microbial communities (pathogens and endosymbionts) of ticks in the United Arab Emirates (UAE) is scarce. Therefore, the aim of the present study was to quantify microbial diversity in *H. anatolicum* using next-generation sequencing (NGS) technology.

**Methods:**

*Hyalomma anatolicum* ticks were collected from livestock in the emirates of Abu Dhabi, Dubai and Sharjah in the UAE during 2019. DNA was extracted from 175 male ticks sampled from livestock (*n* = 78) and subjected to NGS. The* 16S* rRNA gene was analyzed using the Illumina MiSeq platform to determine the bacterial communities. Principal coordinates analysis (PCA) was performed to identify patterns of diversity in the bacterial communities.

**Results:**

Twenty-six bacterial families with high relative abundance were identified, of which the most common were Staphylococcaceae, Francisellaceae, Corynebacteriaceae, Enterobacteriaceae, Moraxellaceae, Bacillaceae, Halomonadaceae, Xanthomonadaceae, Pseudomonadaceae, Enterococcaceae, Actinomycetaceae and Streptococcaceae. The diversity of the microbial communities in terms of richness and evenness was different at the three study locations, with the PCA showing clear clusters separating the microbial communities in ticks collected at Abu Dhabi, Dubai, and Sharjah. The presence of bacterial families harboring pathogenic genera showed that *H. anatolicum* could pose a potential threat to livestock and food security in the UAE.

**Conclusions:**

The study is the first to document important data on the microbial communities associated with *H. anatolicum* in the UAE. This knowledge will facilitate a better understanding of the distribution pattern of microbes in livestock ticks in the UAE and, ultimately, will aid in deciphering the relationships between microbes and in the exploration of potential factors towards developing effective management strategies.

**Graphical Abstract:**

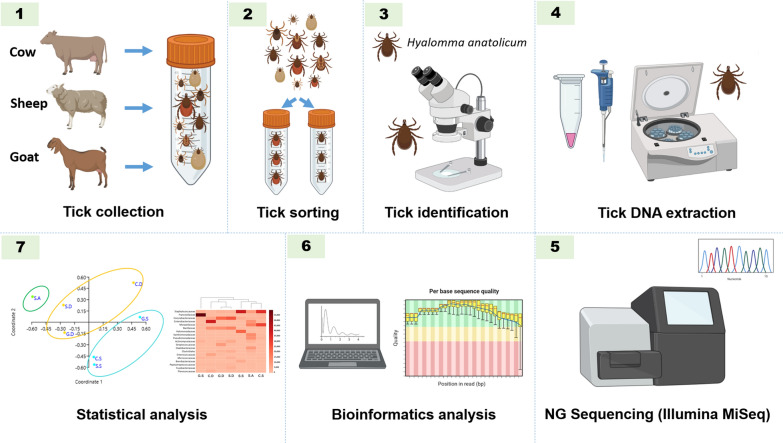

**Supplementary Information:**

The online version contains supplementary material available at 10.1186/s13071-021-05144-z.

## Background

In many areas of the world, tick-borne diseases and tick-related health problems cause significant morbidity in both animals and people. Ticks (Acari: Ixodida) are obligate hematophagous ectoparasites of vertebrates, including humans [1]. They pose a serious threat to the livestock industry by adversely affecting the host’s health through irritation and inflammation on feeding sites, skin damage, anemia, toxicosis and paralysis, or by transmitting a variety of pathogens that cause tick-borne diseases [1, 2]. The ongoing climate change is also a major factor contributing to the spread of tick-borne pathogens in animals and humans across various geographic areas and the emergence or re-emergence of tick-borne diseases [3]. *Hyalomma anatolicum*, a widely distributed tick species that occurs worldwide, is the main vector of piroplasmids, including *Theileria annulata*, *T. lestoquardi*, *T. equi* and *Babesia caballi*, as well as Crimean-Congo hemorrhagic fever virus [4]. In a recent study, *H. anatolicum* in the United Arab Emirates (UAE) was found to be infected with *T. annulata* and *Theileria ovis* [5].

The varied assemblages of pathogenic and non-pathogenic microbes inhabiting the tick gut has effects on the microbes themselves as well as on the host. Ticks harbor a diverse community of microorganisms that could affect vector competence, i.e. the ability of a vector to successfully transmit a pathogen [6, 7]. The interactions between bacterial pathogens and symbionts in tick microbiota remain unresolved. Assemblages of microbes could negatively or positively influence each other, resulting in the suppression or enhancement of some microbial species [7, 8]. In addition, the tick microbiome probably affects the colonization of pathogens within the tick vector and, subsequently, their transmission to hosts [9], possibly altering the epidemiology of tick-borne diseases. For example, removal of the midgut bacteria of *Ixodes scapularis* feeding on antibiotic-treated mice impairs infection by *Borrelia burgdorferi* [10]. The presence of endosymbiotic *Rickettsia bellii* in *Dermacentor andersoni* is associated with lowered infection rates of pathogenic *Anaplasma marginale* [11]. Also, a decrease in *Francisella* endosymbionts is associated with lower *Francisella novicida* infection levels in *Dermacentor andersoni* [11]. Such inter-species interactions suggest that endosymbionts may play an essential role in manipulating the transmission of tick-borne pathogens through effects on microbial community structure [12, 13]. It is still unclear if ticks depend on bacterial endosymbionts to provide vitamins and cofactors that are lacking in the vertebrate host’s blood [14]. However, when *Coxiella*-like bacterium detected in *Amblyomma americanum* ticks [15, 16] were eliminated with antibiotics, there was a severe reduction in tick fecundity and fitness was observed that corresponded with an increase in host fitness [17]. Genomic analysis of the *A. americanum* microbiome communities revealed the metabolic interdependencies of ticks and their nutrient-provisioning endosymbionts, suggesting their potential role as nutritional mutualists [14]. Thus, understanding microbial interactions and their functions at the community level is of vital importance to a better understanding of tick-borne pathogen transmission to animals or humans [9]

Numerous studies have been carried out on the microbiome of ticks infesting livestock [6, 18, 19]. In the UAE, ticks are a continuous problem in livestock [5], particularly in camels. In a study conducted in 2019–2020, the camel tick, *Hyalomma dromedarii*, was found on camels throughout the year [20]. However, to date, only one study has been conducted in the UAE on the bacterial genera present in *H. dromedarii* [8], indicating a lack of research on the microbiomes of several important tick vectors in the country. The microbiomes of ticks could play important roles in the maintenance of tick populations on livestock under unfavorable environmental conditions. Overall, little research has been conducted to quantify tick microbial communities in the UAE. Therefore, the aim of the present study was to quantify microbial diversity in *H. anatolicum* using next-generation sequencing (NGS) technologies. The results will strengthen our understanding of both tick–microbe and microbe–microbe relationships.

## Methods

### Tick collection and identification

The current study had a cross-sectional design. A total of 994 ticks were collected from cows (22), goats (20) and sheep (36) between March and July 2019 from the emirates of Abu Dhabi, Dubai and Sharjah in the UAE. Ticks were collected in sterile 50-ml tubes, placed in an icebox and transported to the Animal Ecology and Entomology Laboratory, UAE University, Al-Ain. Once in the laboratory, the ticks were stored at − 80 °C until DNA extraction. The identification of *H. anatolicum* ticks was confirmed morphologically using taxonomic keys [21, 22].

Tick collection was carried out in strict accordance with the recommendations of the Animal Research Ethics Committee (A-REC) of UAE University (ethical approval no.: ERA_2019_5953). The experimental protocol was also approved by the UAE University Research Office.

### Genomic DNA extraction and pooling

Only male *H. anatolicum* were selected for DNA extraction due to an insufficient number of female ticks. Before DNA extraction, ticks were washed in 70% ethanol and deionized water for 5 min to remove environmental contaminants in accordance with a published protocol [23]. Due to the small size of each tick, a pool of five male *H. anatolicum* ticks was homogenized in liquid nitrogen inside a sterile 1.5-ml microcentrifuge tube using a sterile Kimble Kontes pellet pestle (Thermo Fisher Scientific, Waltham, MA, USA). Genomic DNA was extracted from each pool using the QIAamp Tissue Kit (Qiagen, Hilden, Germany) following the manufacturer’s protocol. The concentration and quality of DNA were assessed using a Nano Drop ND-100 spectrophotometer (Peqlab Biotechnologie GmbH, Erlangen, Germany). DNA quality was also determined by electrophoresis in a 1% agarose gel; the bands were stained with ethidium bromide and visualized under UV light. DNA was stored at − 20 °C in the freezer until further use. Prior to sequencing, extracted DNA samples (5 DNA samples of ticks from each host) were pooled again to make one pool for each host from each location, resulting in seven DNA pools.

### Next-generation sequencing and bioinformatics analysis

*16S* ribosomal RNA (rRNA) gene-based analysis was conducted to determine the composition of the bacterial communities in *H. anatolicum*. For NGS, each of the seven DNA pools were sequenced by Macrogen Inc. (Seoul, South Korea). A pair of primers, Bakt_341F (CCT ACG GGNGGC WGC AG) and Bakt_805R (GAC TAC HVGGG TAT CTA ATC C [24]), was used to amplify the hypervariable V3–V4 region of the* 16S* rRNA gene. PCR was performed using the Herculase II Fusion DNA polymerase Nextera XT Index Kit V2 (Agilent Technologies, Inc., Santa Clara, CA, USA), and sequencing was performed on an Illumina MiSeq platform (Illumina, Inc., San Diego, CA, USA) with a read length of 301 bp. Fast length adjustment of short reads (FLASH) version 1.2.11 [25] was used to merge FASTQ paired-end sequences. Read quality by sample is given in Additional file 1: Table S1. Next, merged reads were clustered into operational taxonomic units (OTUs) using CD-HIT-OTU [26] with default options. The CD-HIT-OTU workflow includes several preprocessing steps and filters low-quality reads, trims long tails, identifies chimeric reads and, finally, clusters the reads into OTUs with an identity cutoff of 97%. Finally, the assign_taxony.py script from QIIME1.9.1 [27] was used for the taxonomic assignment of OTUs. The assignment was based on Basic Local Alignment Search Tool (BLAST) [28] searches in the National Center for Biotechnology Information (NCBI)* 16S* microbial database and the Ribosomal Database Project (RDP; http://rdp.cme.msu.edu/). Taxonomic abundance count was aggregated in Microsoft Excel (Microsoft Corp., Redmond, WA, USA) to calculate abundance ratios at the phylum, class, family and genus levels.

### Statistical analyses

Principal Coordinates Analysis (PCoA) was conducted to determine diversity patterns in bacterial communities in *H. anatolicum* using the Paleontological Statistics Software Package (PAST) 5.27 [29]; the OTU count for each genus was entered and the samples were classified by locations (Abu Dhabi, Dubai, and Sharjah). We examined the Eigenvalues to determine the magnitude of variation [30]. Different indices of diversity were calculated since a single index often does not reflect the true nature of diversity and a combination provides a better approximation of diversity. Richness (total number of genera, based on OTUs obtained for each genus), the Shannon–Wiener Index and the Index of Dominance were estimated. Pearson’s correlation coefficient (*r*) was calculated to determine the associations between different genera [31]. Stepwise regression analysis was performed with backward selection with the genera that only showed significant correlations [31]. The value of *α* was set at 0.05 for all tests.

## Results

### Bacterial* 16S* rRNA gene abundance profile

A total of 476,949 read counts were obtained (range 51,680–83,201 reads, average 68,135.571 reads). The raw reads were subsequently used for taxonomic classification after the sequences were subjected to quality filtering. A total of 372 OTUs were generated through the de novo picking process (clustered at 97% similarity), belonging to 14 phyla, 22 classes, 81 families and 153 genera.

### Relative abundance of bacteria

Taxonomic profiling of the microorgansms collected from *H. anatolicum* resulted in the identification of eight phyla that were abundant: Proteobacteria, Firmicutes, Actinobacteria, Bacteroidetes, Fusobacteria, Cynobacteria, Planctomycetes and Chloroflexi. Of these Proteobacteria and Firmicutes were the most abundant phyla, and Chloroflexi and Planctomycetes were the least abundant (Additional file 2: Table S2).

Ten bacterial classes of the 22 identified were abundant: Gammaproteobacteria, Bacilli, Actinobacteria, Clostridia, Alphaproteobacteria, Betaproteobacteria, Fusobacteriia, Flavobacteriia, Erysipelotrichia and Bacteroidia. Gammaproteobacteria was recorded as the most dominant class in ticks collected from cows (from Dubai), goats (from Sharjah), and sheep (from Abu Dhabi), while Bacilli was found to be the most dominant class in ticks collected from sheep from Dubai and Sharjah, cows from Sharjah and goats from Dubai (Additional file 3: Table S3).

**Fig. 1:**
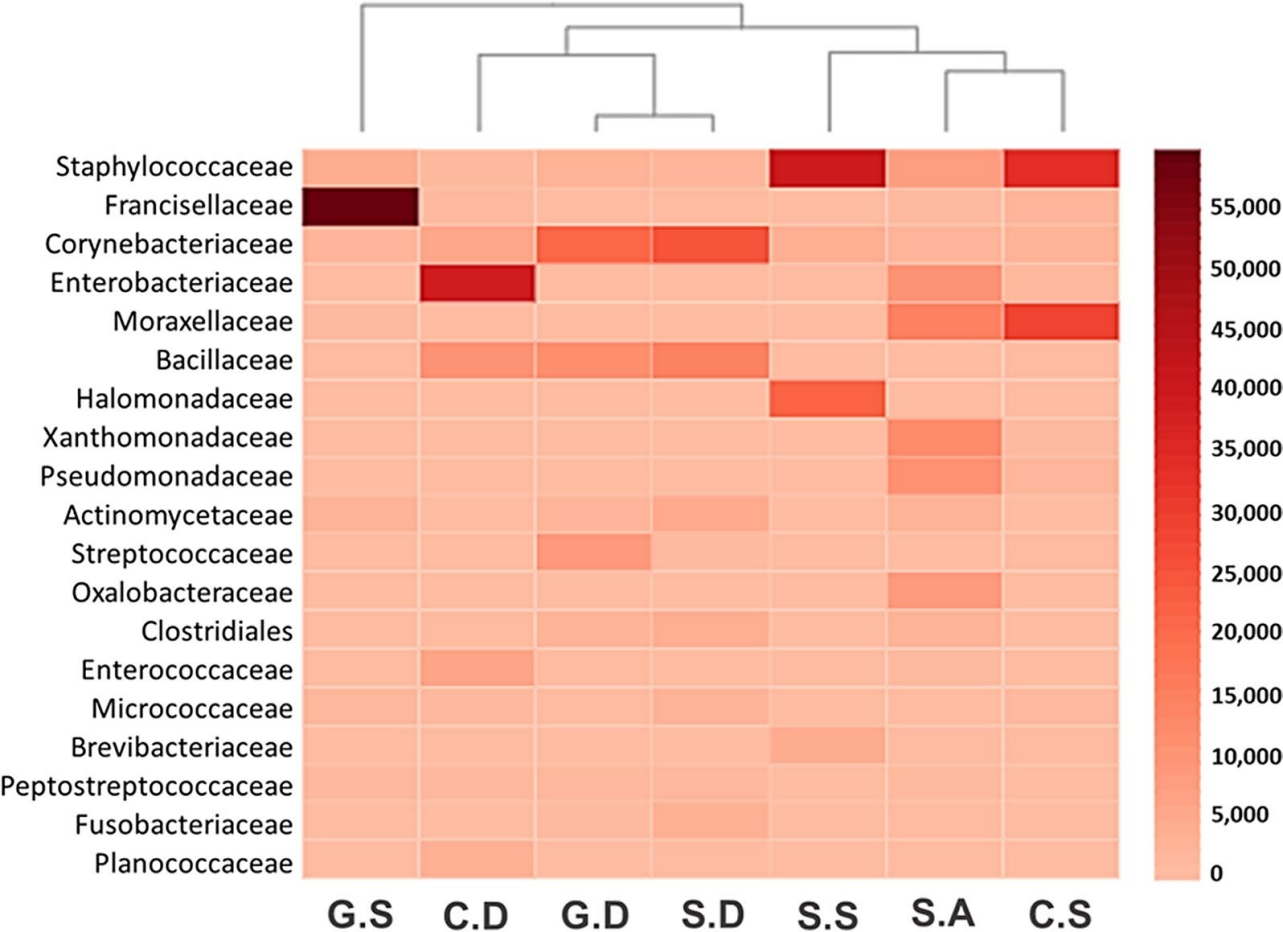
Microbial families detected in *H. anatolicum* adult male ticks collected from goats, cows and sheep in three emirates of the United Arab Emirates in 2019 and 2020. Abbreviations: G.S, Goats, Sharjah; C.D, cows, Dubai; G.D, goats, Dubai; S.D, sheep, Dubai; S.S, sheep, Sharjah; S.A, sheep, Abu Dhabi; C.S, cows, Sharjah

Taxonomic assignment revealed that 26 bacterial families were abundant (Additional file 3: Table S3), among which the following showed the highest relative abundance: Francisellaceae (72%) detected in ticks collected from goats, Enterobacteriaceae (57.9%) and Moraxellaceae (38.8%) detected in ticks from cows and Staphylococcaceae (57.7%), Corynebacteriaceae (41.5%), Halomonadaceae (31.7%) and Bacillaceae (23.6%) detected in ticks from sheep (Fig. 1; Additional file 4: Table S4).

**Fig. 2:**
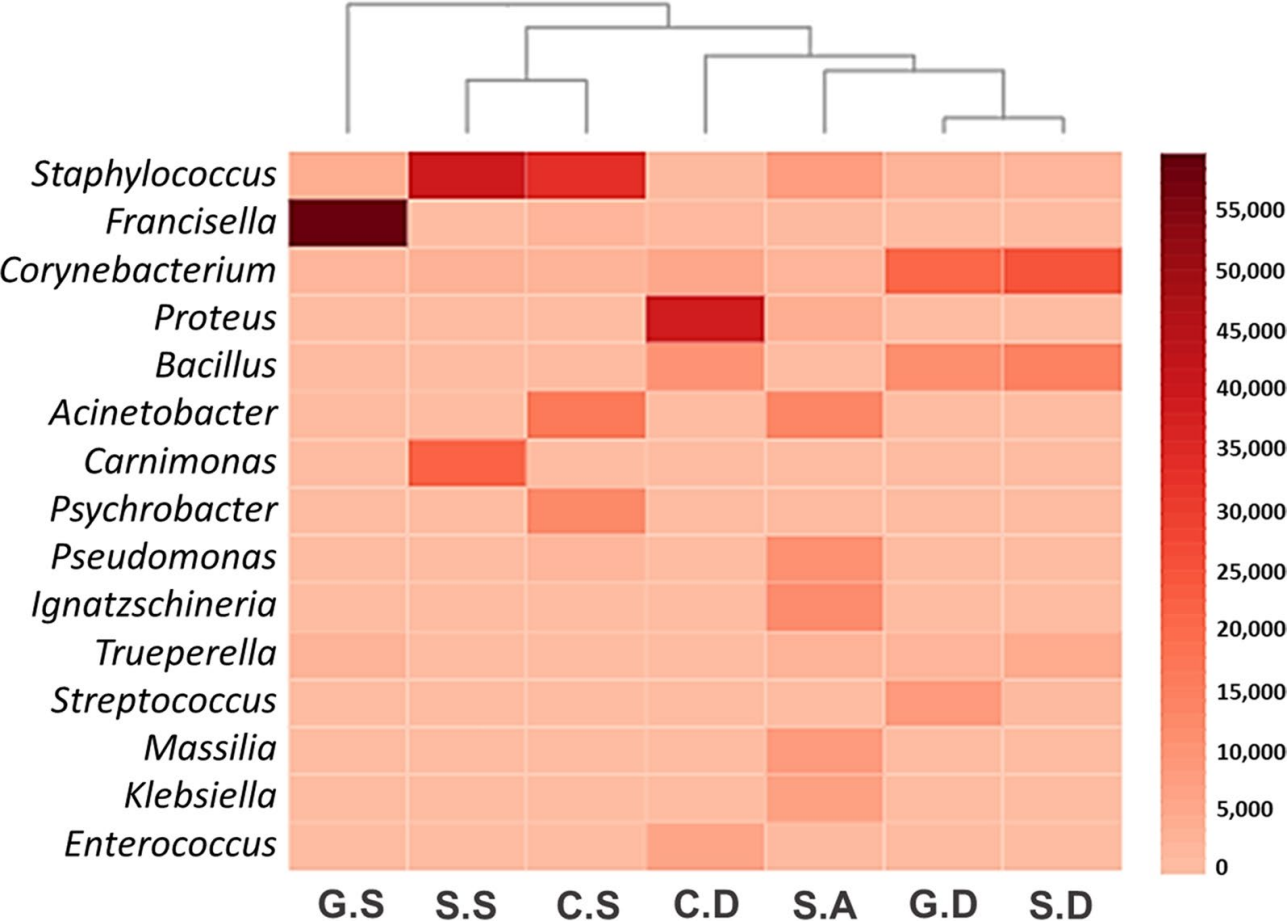
Microbial genera detected in *H. anatolicum* adult male ticks collected from goats, cows and sheep in three emirates of the UAE in 2019 and 2020. Abbreviations as in Fig. 1 caption

In the microbiome of *H. anatolicum*, the relative abundance of genera was highly variable among all hosts.* Staphylococcus* and* Corynebacterium* were the two most common genera reported from all locations, with high relative abundance detected in sheep (57.6% and 41.5%, respectively). *Francisella* had a relative abundance of 72% in ticks collected from goats, followed by *Proteus* (57.9%) from cows and *Carnimonas* (31.7%) and *Bacillus* (23.6%) from sheep. *Acinetobacter*, *Psychrobacter*, *Ignatzschineria, Streptococcus*, *Pseudomonas*, *Massilia*, *Klebsiella*, *Trueperella* and *Enterococcus* occurred at moderately low relative abundances (6–22%). However, the genera *Brevibacterium*, *Fusobacterium*, *Enterobacter*, *Peptoniphilus*, *Nesterenkonia*, *Peptostreptococcus*, *Wautersiella*, *Murdochiella*, *Clostridium*, *Alloiococcus*, *Turicella*, *Arthrobacter*, *Propionibacterium*, *Helcococcus*, *Sporosarcina, Parvimonas*, *Anaerococcus*, *Salmonella*, *Enhydrobacter* and *Auritidibacter* were all found to be present at low relative abundance (1–5.4%) (Fig. 2; Additional file 5: Table S5).

### Microbial richness and evenness

**Fig. 3:**
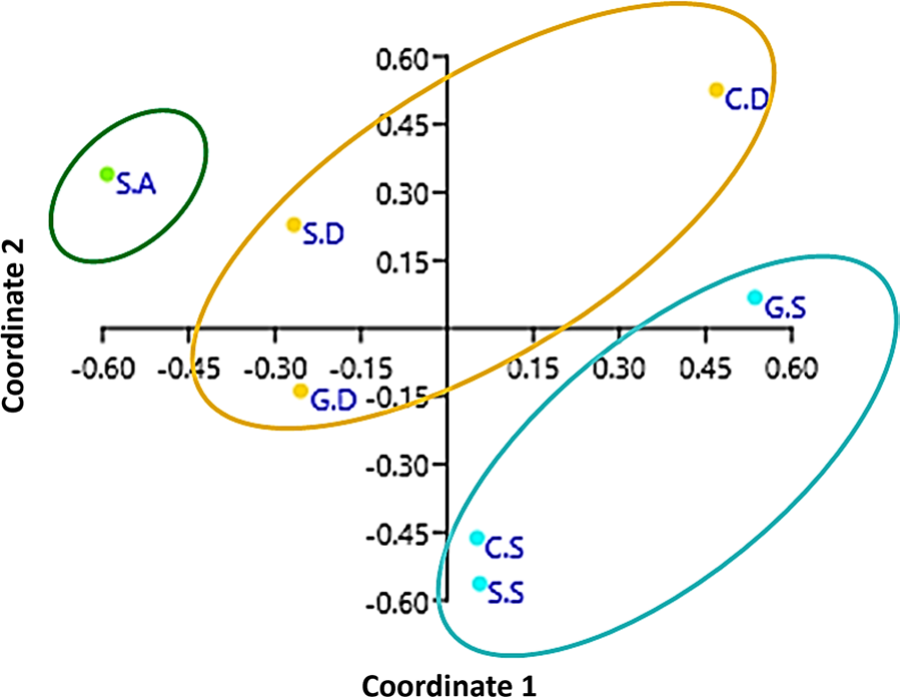
Principal coordinates analysis showing microbial diversity between study locations. Filled blue, gold and green circles refer to the emirates of Sharjah, Dubai and Abu Dhabi, respectively. Abbreviations as in Fig. 1 caption

Principal coordinates analysis showed that coordinates 1, 2, 3 and 4 accounted for > 84% of the variation (based on cumulative Eigenvalues), with the first two coordinates accounting for > 54% of the variation. There was also a clear separation of the microbial communities according to location (Fig. 3). The richness of genera differed significantly between cow samples from Dubai and Sharjah (4.24 in cows, Dubai [C.D] versus 1.56 in cows, Sharjah [C.S]; two-sample paired t-test, *P* < 0.01). The Shannon–Wiener index differed significantly between C.D and C.S (1.118 [95% confidence interval: 1.111–1.127] vs 1.308 [95% confidence interval: 1.302–1.315], respectively; two sample paired t-test, *P* < 0.05). The Index of Evenness was significantly lower  in C.D than in C.S (0.25 vs 0.37, respectively; two-sample paired t-test, *t* = − 35.747, *P* < 0.01). The Index of Dominance was also significantly different between C.D and C.S (0.45 vs 0.33, respectively; two-sample paired t-test, *P* < 0.01). Similarly, the richness of genera differed significantly between goat samples from Dubai and Sharjah (2.13 in goats, Dubai[(G.D] vs 4.34 in goats, Sharjah [G.S]; two-sample paired t-test, *P* < 0.01). The Index of Evenness was significantly higher  in G.D than in G.S (0.344 vs 0.139, respectively; two-sample paired t-test, *P* < 0.01). The Index of Dominance was significantly different between G.D and G.S (0.32 vs 0.78, respectively; two-sample paired t-test, *P* < 0.01). Further, the richness and evenness of genera were significantly different between sheep samples from Abu Dhabi, Dubai and Sharjah.

### Associations between bacterial genera

**Fig. 4:**
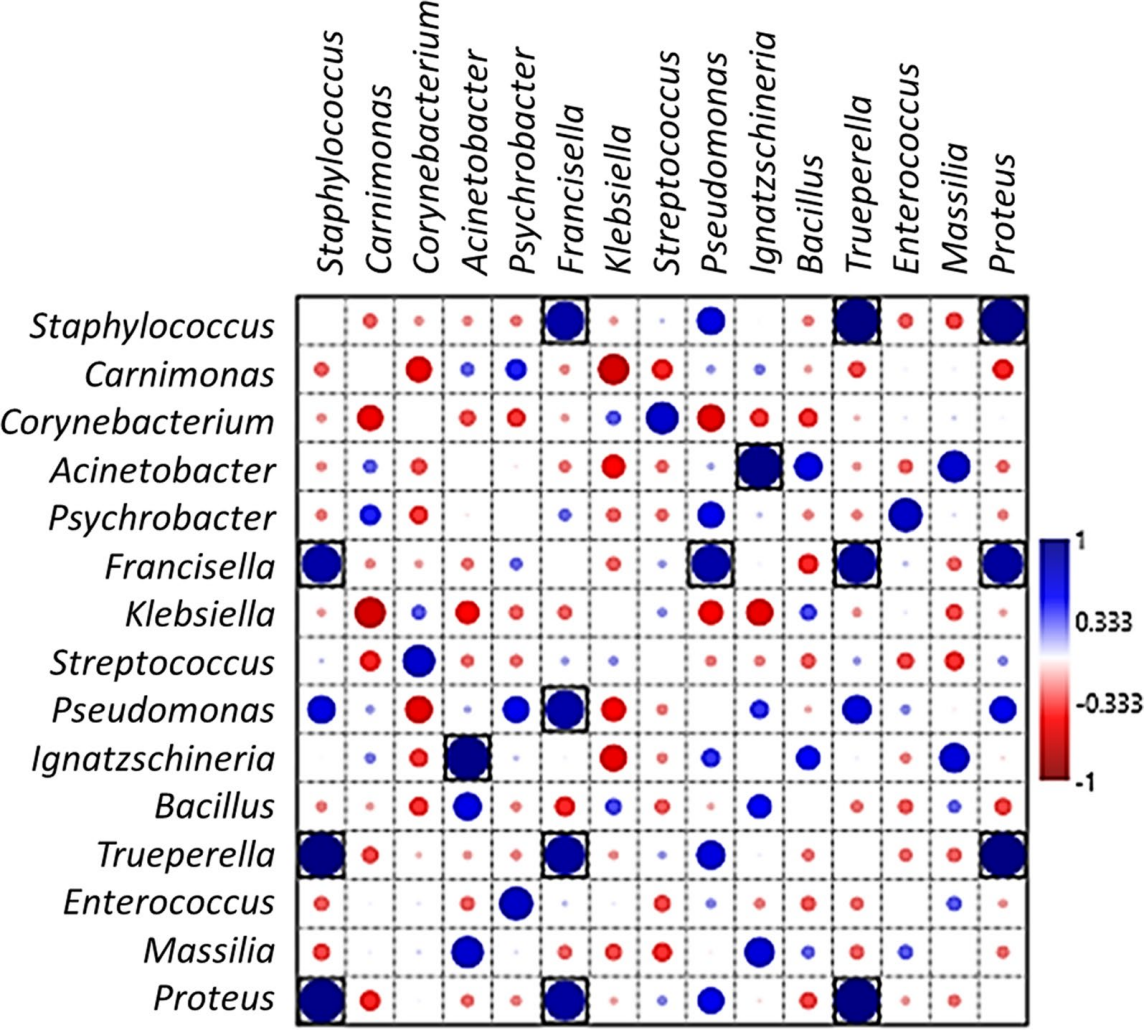
Pearson’s correlation coefficients indicating associations between bacterial genera showing significantly positive interactions (large dark-blue circles) and significantly negative interactions (large red circles). Black boxes denote *P* < 0.05

Pearson’s correlation coefficients (*r*) indicated that several microbial genera were significantly correlated with each other (*P* < 0.05, boxed circles) (Fig. 4; Additional file 6: Table S6). *Fransicella* was significantly positively correlated with *Pseudomonas*, *Trueperella* and *Proteus*; *Acinetobacter* and *Enterococcus* were positively correlated with some of the other genera. However, *Klebsiella* was negatively correlated with *Carnimonas*, *Ignatzschineria, Pseudomonas,* and *Acinetobacter*. In addition, *Corynebacterium* was negatively correlated with *Carnimonas* and *Pseudomonas.* We did not find significant relationships between response variables and explanatory variables.

## Discussion

Overall, our results show that *Staphylococcus*, *Francisella*, *Corynebacterium*, *Proteus* and *Bacillus* were the most abundant microbial genera in *H. anatolicum*; other genera were also present but at lower levels. Thus, this study provides a snapshot of the internal microbiome and sheds light on the potential tick-borne pathogens and endosymbionts associated with *H. anatolicum* in the UAE. Previous studies showed that vector-borne infections in the vertebrate host shape the microbiome of the arthropod vector and their competence to acquire and maintain infections with vector-borne pathogens [32]. Therefore, the results of the current study will help increase our understanding of disease dynamics in livestock. Our observation that *Staphylococcus* and *Francisella* were the most common genera in *H. anatolicum* indicates that some pathogenic species, such as *Staphylococcus aureus* or *Francisella tularensis*, may be present in the animal population this tick species parasitizes.

We obtained a lower number of bacterial reads and OTUs (372) from male *H. anatolicum* as compared to earlier studies which reported 1314 OTUs (sex of tick is not mentioned [6]) and 6023 OTUs (female *H. anatolicum* [33]) on the microbiome of *H. anatolicum*. This difference can probably be attributed to differences in the life-cycle due to the geographical separation of species and climatic conditions of the Middle East and Asia. Furthermore, our results support the observations from earlier *Hyalomma* tick microbiome studies regarding bacterial phyla. For example, the pattern of bacterial phyla presence in the current study is consistent with that reported in earlier studies in which Proteobacteria, Firmicutes and Actinobacteria were found to be abundant in *H. anatolicum* [6] and *H. dromedarii* [8]. In addition, we found Gammaproteobacteria and Bacilli to be dominant classes in *H. anatolicum* ticks collected from all three hosts, as reported earlier [19], however their relative abundance varied among the three sampling locations of the UAE.

In an earlier study we found that the bacterial family Francisellaceae was present in very high relative abundance (99.1%) in the camel tick *H. dromedarii* [8]; in the present study we similarly detected Francisellaceae with high relative abundance (72%) in *H. anatolicum* collected from goats (Fig. 1). In addition, we observed Enterobacteriaceae and Moraxellaceae to be present with high relative abundance (57.9% and 38.8%, respectively) in *H. anatolicum* collected from cows, which differs from the results of some other studies [19] that reported Staphylococcaceae, Oxalobacteraceae, Burkholderiaceae and Pseudomonades as dominant families in *H*. *anatolicum* collected from cattle. However, we detected a high relative abundance of Staphylococcaceae (57.7%), Corynebacteriaceae (41.5%), Halomonadaceae (31.7%) and Bacillaceae (23.6%) from sheep samples. The composition and diversity of bacterial families inside each individual tick were probably influenced by external and stochastic factors [8]. Further, tick microbial diversity could also be affected by host species and host species’ competence to sustain different types of microbes.

In total, 153 genera were identified in *H. anatolicum*. Interestingly, *Staphylococcus* and *Corynebacterium* were present in almost all locations and, in addition, both genera were detected with high relative abundance (57.6%) and (41.5%) in ticks collected from sheep. Similar results have been reported previously, where *Corynebacterium* and *Staphylococcus* were the most dominant genera in tick species [19, 34]; it is likely ticks acquire these microorganisms from livestock skin and fur. In the current study, *Francisella* was detected with a high relative abundance of 72% in ticks collected from goats, followed by *Proteus* (57.9%) from cows. Recently, a high prevalence of endosymbionts, such as *Francisella*-like endosymbionts (91.5%), has been reported in bovine ticks [18]; however, a previous study [19] reported *Francisella* with only 0.2% abundance in *H. anatolicum* ticks collected from buffaloes during a study exploring the microbiomes of ticks collected from different species of livestock from Pakistan. However, in the UAE, the genus *Francisella* was previously reported in *H. dromedarii* collected from camels, with a high relative abundance of 99.1% [8]. In addition, almost similar results were reported from Saudi Arabia, where *Francisella* was found to be a dominant genus with an abundance of 94.4% [34] and 42.1% [35] in *H. dromedarii*. It has been shown that tick endosymbionts mostly belong to the genera *Francisella, Rickettsia* and *Coxiella* [36] and that they probably have mutualistic relationships with ticks possibly also modulate tick vector capacity by influencing tick-borne pathogens’ colonization and transmission to vertebrate hosts [9, 37]. In addition, bacterial endosymbionts of ticks might influence survival, fitness and reproduction, nutritional adaptation and immunity [9, 13]. The endosymbionts may vary significantly among tick species across the world, and their composition among tick species is affected by numerous factors, such as tick species [38, 39], tick developmental stage [40], feeding status of tick [41], co-existence of pathogens inside ticks [37], environmental conditions [41], seasonality [38], and geographical area [39]. Although the genera *Francisella, Rickettsia* and *Coxiella* might be present as endosymbionts in some tick species, including *H. anatolicum*, this does not rule out the possibility of the presence of some of their pathogenic species in a few tick populations now or in the future in the UAE. Therefore, this study underlines the importance of conducting disease screening programs to ensure the early detection of tick-borne pathogens. Additionally, our analysis indicated the presence of few read counts for the genus *Ehrlichia* in tick samples collected from cows from Dubai and Sharjah and goats from Dubai. However, Ghafar et al. [18] recently reported high abundance of *Ehrlichia* in *H. anatolicum* ticks collected from livestock. These differences might be linked to different factors between the habitats of the two studies. In general, mixed infections in ticks, such as *Francisella*-like endosymbionts and piroplasmids and/or *Ehrlichia* spp., may exist in ticks in the UAE. It should be mentioned that ticks which are co-infected with multiple pathogens might pose a serious threat to animals and humans, which may intensify the clinical complexity of diseases [42]. Thus, studies are required for large-scale PCR screening of pathogens, such as *Ehrlichia*, to assess their interaction with other pathogens/endosymbionts as well as its zoonotic potential.

Overall, we found significant positive associations between *Fransicella* and *Pseudomonas,* as well as between *Trueperella,* and *Proteus*. Thus far, little is known about *Francisella* spp. and their associations with other microbes, and how they shape the tick microbiome and alter the tick-borne disease epidemiology. Studies have confirmed that symbiotic bacterial genera co-exist with pathogenic ones [12] and endosymbiotic forms under shared environmental preferences, could have significant patterns of positive or negative co-occurrence with pathogenic forms and could facilitate, limit or block pathogen transmission, depending on the nature of tick microbial interactions [12, 43]. The genus *Francisella* has been reported in *H. dromedarii* in the UAE [8], and it likely to be found as an endosymbiont. However, globally, the phylogenetic similarities between endosymbiont and pathogenic forms suggest periodic and perhaps even frequent shifts from non-pathogenic to pathogenic forms [12]. Noteworthy, *Trueperella* species are opportunistic pathogens and considered to be a part of the biota of the skin and upper respiratory and urogenital tracts of cattle, goats and sheep; however, its reservoirs and transmission routes and pathogenesis are poorly understood [44]. In the present study, we found *Trueperella* with low abundance in goats and sheep samples (Fig. 2). In addition, we detected *Pseudomonas* in sheep, which is also an opportunistic pathogen that can be found in a variety of soils [45]. However, Brown et al. [46] found that adult *Dermacentor andersoni* ticks may be killed by *Proteus mirabilis*. Furthermore, we found a negative association between *Klebsiella* and *Carnimonas*, *Ignatzschineria, Pseudomonas* and *Acinetobacter.* Interestingly, *Acinetobacter* spp. occupy diverse environments, such as soil and freshwater, and many are known to be pathogenic, while others are considered to be commensal and part of the normal animal flora [47]. It is known that ticks can acquire microbiota of skin/fur during feeding on the host, while the microbes present in the vegetation or soil probably colonize ticks on the ground when they drop off their vertebrate hosts [48]. However, tick-borne infections in the vertebrate host shape the microbiome of ticks [32]. We suggest that the five abovementioned bacterial genera need further confirmation through PCR screening using genus-specific primers. In addition, continuous surveillance and screening of bacterial genera is important for the development of mitigation strategies for tick-borne pathogens.

## Conclusions

In this study, we quantified the internal microbiome present in male *H. anatolicum*. The findings of the current study will fuel interest to improve our understanding of the distribution pattern of microbes and to visualize the pathogens and symbionts in the microbial fauna of *H. anatolicum* and other livestock ticks in the UAE. *Staphylococcus*, *Francisella*, *Corynebacterium*, *Proteus*, and *Bacillus* were the most abundant microbial genera identified in this study. This work represents an initial step towards enhancing future comparative microbiome studies in the UAE and elsewhere. Nonetheless, it remains essential to mention that further research is required to investigate and screen samples using species-specific primers for the characterization of endosymbionts and tick-borne pathogens.

## Supplementary Information

**Additional file 1: Table S1.** Read quality by sample.

**Additional file 2: Table S2.** Microbial phyla (presence in %) detected in *H. anatolicum* adult ticks from three emirates in the UAE.

**Additional file 3: Table S3.** Microbial classes (presence in %) detected in *H. anatolicum* adult ticks from three emirates in the UAE.

**Additional file 4: Table S4.** Microbial families (presence in %) detected in *H. anatolicum* adult ticks from three emirates in the UAE.

**Additional file 5: Table S5.** Microbial genera (presence in %) detected in *H. anatolicum* adult ticks from three emirates in the UAE.

**Additional file 6: Table S6.** Correlation matrix showing pairwise Pearson’s *r* correlations between genera (bottom) and their associated significance (top).

## Data Availability

All data generated or analyzed during this study are included in this published article.

## Abbreviations

C.D: Cows, Dubai
C.S: Cows, Sharjah
G.D: Goats, Dubai
G.S: Goats, Sharjah
S.A: Sheep, Abu Dhabi
S.D: Sheep, Dubai
S.S: Sheep, Sharjah
UAE: United Arab Emirates
