# Supplementary material for: Microbial composition in Hyalomma anatolicum collected from livestock in the United Arab Emirates using next-generation sequencing
Source: Parasit Vectors. 2022 Jan 20;15:30. doi: 10.1186/s13071-021-05144-z (PMC8772180; doi:10.1186/s13071-021-05144-z)
Supplement: Supplementary file 1 — Additional file 1: Table S1. Read quality by sample. [file 13071_2021_5144_MOESM1_ESM.docx]

**Additional file 1: Table S1.** Read quality by sample

| Sample Name | Total Bases | Read Count | N (%) | GC (%) | Q20 (%) | Q30 (%) |
| --- | --- | --- | --- | --- | --- | --- |
| Cow Dubai | 67,630,846 | 146,633 | 0.0005 | 54.1 | 98.44 | 94.96 |
| Cow Sharjah | 66,232,172 | 142,966 | 0.0005 | 51.91 | 98.52 | 95.25 |
| Goat Dubai | 61,972,746 | 137,255 | 0.0005 | 55.19 | 98.42 | 94.6 |
| Goat Sharjah | 65,451,099 | 142,636 | 0.0006 | 51.45 | 98.6 | 95.4 |
| Sheep Abu Dhabi | 72,233,970 | 156,403 | 0.0006 | 52.74 | 98.47 | 95.06 |
| Sheep Dubai | 61,805,685 | 136,564 | 0.0004 | 54.84 | 98.3 | 94.29 |
| Sheep Sharjah | 71,969,390 | 155,268 | 0.0005 | 53.34 | 98.59 | 95.35 |
| Total Bases : The total number of bases in reads identified Read Count : The total number of sequence reads N(%) : The N percentage in sequence reads GC(%) : The GC percentage in sequence reads Q20(%) : The percentage of bases in which the Phred score is above 20 Q30(%) : The percentage of bases in which the Phred score is above 30 | | | | | | |
