# Supplementary material for: Microbial composition in Hyalomma anatolicum collected from livestock in the United Arab Emirates using next-generation sequencing
Source: Parasit Vectors. 2022 Jan 20;15:30. doi: 10.1186/s13071-021-05144-z (PMC8772180; doi:10.1186/s13071-021-05144-z)
Supplement: Supplementary file 2 — Additional file 2: Table S2. Microbial phyla (presence in %) detected in H. anatolicum adult ticks from three emirates in the UAE. [file 13071_2021_5144_MOESM2_ESM.docx]

**Additional file 3: Table S3.** Microbial classes (presence in %) detected in *H. anatolicum* adult ticks from three emirates in UAE.

| Class | C.D | C.S | G.D | G.S | S.A | S.D | S.S |
| --- | --- | --- | --- | --- | --- | --- | --- |
| Gammaproteobacteria | 59.01% | 45.15% | 0.26% | 72.99% | 65.37% | 0.31% | 31.84% |
| Alphaproteobacteria | 0.21% | 0.80% | 0.29% | 9.28% | 0.18% | 0.15% | 0.20% |
| Actinobacteria | 9.51% | 5.35% | 44.24% | 6.49% | 6.01% | 53.46% | 9.99% |
| Bacilli | 28.22% | 46.77% | 43.59% | 5.13% | 11.42% | 30.72% | 57.88% |
| Clostridia | 2.39% | 0.15% | 10.20% | 2.61% | 5.02% | 10.11% | 0.02% |
| Bacteroidia | 0.00% | 0.01% | 0.12% | 0.85% | 0.36% | 0.17% | 0.00% |
| Erysipelotrichia | 0.39% | 0.09% | 0.14% | 0.73% | 0.03% | 0.42% | 0.04% |
| Deltaproteobacteria | 0.00% | 0.00% | 0.00% | 0.36% | 0.00% | 0.00% | 0.00% |
| Negativicutes | 0.00% | 0.00% | 0.00% | 0.19% | 0.00% | 0.01% | 0.00% |
| Cyanobacteria | 0.00% | 0.00% | 0.00% | 0.14% | 0.00% | 0.00% | 0.00% |
| Planctomycetia | 0.00% | 0.00% | 0.00% | 0.13% | 0.00% | 0.00% | 0.00% |
| Betaproteobacteria | 0.00% | 0.00% | 0.04% | 0.06% | 11.31% | 0.00% | 0.00% |
| Verrucomicrobiae | 0.00% | 0.00% | 0.00% | 0.02% | 0.00% | 0.00% | 0.00% |
| Sphingobacteriia | 0.00% | 0.00% | 0.00% | 0.00% | 0.01% | 0.00% | 0.00% |
| Methanobacteria | 0.03% | 0.00% | 0.00% | 0.00% | 0.00% | 0.00% | 0.00% |
| Acidobacteria_Gp3 | 0.00% | 0.02% | 0.00% | 0.00% | 0.00% | 0.00% | 0.00% |
| Flavobacteriia | 0.01% | 1.61% | 0.00% | 0.00% | 0.09% | 0.00% | 0.00% |
| Thermomicrobia | 0.13% | 0.00% | 0.03% | 0.00% | 0.00% | 0.01% | 0.00% |
| Deinococci | 0.01% | 0.00% | 0.00% | 0.00% | 0.00% | 0.00% | 0.00% |
| Fusobacteriia | 0.00% | 0.00% | 0.98% | 0.00% | 0.21% | 4.57% | 0.01% |
| Epsilonproteobacteria | 0.00% | 0.00% | 0.01% | 0.00% | 0.00% | 0.00% | 0.00% |
| Chloroplast | 0.02% | 0.04% | 0.08% | 0.00% | 0.00% | 0.05% | 0.00% |
