# Supplementary material for: Microbial composition in Hyalomma anatolicum collected from livestock in the United Arab Emirates using next-generation sequencing
Source: Parasit Vectors. 2022 Jan 20;15:30. doi: 10.1186/s13071-021-05144-z (PMC8772180; doi:10.1186/s13071-021-05144-z)

**Additional file 6: Table S6.** Correlation matrix showing pairwise Pearson’s r correlations between genera (bottom) and their associated significance (top).


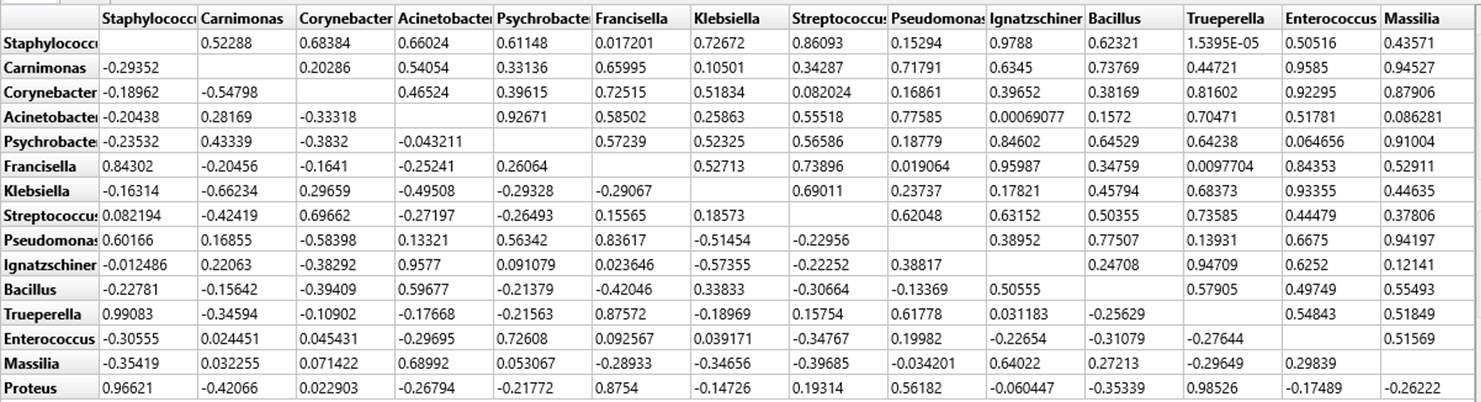

Supplement: Supplementary file 3 — Additional file 3: Table S3. Microbial classes (presence in %) detected in H. anatolicum adult ticks from three emirates in the UAE. [file 13071_2021_5144_MOESM3_ESM.docx]
