# Supplementary material for: Microbial composition in Hyalomma anatolicum collected from livestock in the United Arab Emirates using next-generation sequencing
Source: Parasit Vectors. 2022 Jan 20;15:30. doi: 10.1186/s13071-021-05144-z (PMC8772180; doi:10.1186/s13071-021-05144-z)
Supplement: Supplementary file 4 — Additional file 4: Table S4. Microbial families (presence in %) detected in H. anatolicum adult ticks from three emirates in the UAE. [file 13071_2021_5144_MOESM4_ESM.docx]

**Additional file 4: Table S4.** Microbial families (presence in %) detected in *H. anatolicum* adult ticks from three emirates in UAE.

| Family | C.D | C.S | G.D | G.S | S.A | S.D | S.S |
| --- | --- | --- | --- | --- | --- | --- | --- |
| Staphylococcaceae | 1.15% | 45.38% | 4.36% | 4.27% | 10.48% | 2.80% | 57.71% |
| Moraxellaceae | 0.02% | 38.78% | 0.13% | 0.46% | 20.10% | 0.02% | 0.10% |
| Corynebacteriaceae | 7.44% | 2.68% | 40.33% | 2.02% | 2.43% | 41.53% | 4.49% |
| Francisellaceae | 1.02% | 2.26% | 0.09% | 72.01% | 0.53% | 0.22% | 0.02% |
| Pseudomonadaceae | 0.02% | 1.98% | 0.00% | 0.01% | 14.47% | 0.00% | 0.00% |
| Flavobacteriaceae | 0.01% | 1.61% | 0.00% | 0.00% | 0.09% | 0.00% | 0.00% |
| Enterobacteriaceae | 57.94% | 1.45% | 0.04% | 0.11% | 13.81% | 0.08% | 0.00% |
| Micrococcaceae | 1.13% | 0.90% | 0.29% | 1.44% | 0.09% | 3.44% | 0.08% |
| Aerococcaceae | 0.07% | 0.64% | 1.80% | 0.00% | 0.27% | 0.69% | 0.00% |
| Xanthomonadaceae | 0.03% | 0.56% | 0.00% | 0.08% | 16.43% | 0.00% | 0.00% |
| Brevibacteriaceae | 0.07% | 0.48% | 0.04% | 0.06% | 0.40% | 0.01% | 5.41% |
| Streptococcaceae | 0.17% | 0.32% | 15.60% | 0.00% | 0.04% | 0.66% | 0.00% |
| Bacillaceae | 14.58% | 0.24% | 21.12% | 0.20% | 0.00% | 23.64% | 0.00% |
| Enterococcaceae | 8.16% | 0.10% | 0.58% | 0.03% | 0.59% | 0.00% | 0.13% |
| Erysipelotrichaceae | 0.39% | 0.09% | 0.14% | 0.73% | 0.03% | 0.42% | 0.04% |
| Peptostreptococcaceae | 1.30% | 0.07% | 1.97% | 1.09% | 0.73% | 1.84% | 0.02% |
| Clostridiaceae | 0.36% | 0.03% | 0.10% | 0.70% | 0.04% | 0.14% | 0.00% |
| Halomonadaceae | 0.00% | 0.02% | 0.00% | 0.01% | 0.02% | 0.00% | 31.71% |
| Ruminococcaceae | 0.05% | 0.02% | 0.02% | 0.20% | 0.04% | 1.48% | 0.00% |
| Planococcaceae | 3.98% | 0.01% | 0.02% | 0.02% | 0.04% | 0.25% | 0.00% |
| Carnobacteriaceae | 0.00% | 0.00% | 0.10% | 0.31% | 0.01% | 2.24% | 0.03% |
| Clostridiales | 0.35% | 0.00% | 3.89% | 0.01% | 2.99% | 5.39% | 0.00% |
| Incertae Sedis | 0.00% | 0.00% | 3.82% | 0.00% | 0.46% | 0.63% | 0.00% |
| Actinomycetaceae | 0.10% | 0.00% | 3.25% | 2.72% | 3.05% | 7.13% | 0.00% |
| Porphyromonadaceae | 0.00% | 0.00% | 0.09% | 0.64% | 0.27% | 0.04% | 0.00% |
| Oxalobacteraceae | 0.00% | 0.00% | 0.04% | 0.03% | 11.01% | 0.00% | 0.00% |
| others | 0.12% | 0.62% | 0.03% | 9.03% | 0.11% | 0.03% | 0.17% |
