# Supplementary material for: Microbial composition in Hyalomma anatolicum collected from livestock in the United Arab Emirates using next-generation sequencing
Source: Parasit Vectors. 2022 Jan 20;15:30. doi: 10.1186/s13071-021-05144-z (PMC8772180; doi:10.1186/s13071-021-05144-z)
Supplement: Supplementary file 5 — Additional file 5: Table S5. Microbial genera (presence in %) detected in H. anatolicum adult ticks from three emirates in the UAE. [file 13071_2021_5144_MOESM5_ESM.docx]

**Additional file 5: Table S5.** Microbial genera (presence in %) detected in *H. anatolicum* adult ticks from three emirates in UAE.

| Genus | C.D | C.S | G.D | G.S | S.A | S.D | S.S |
| --- | --- | --- | --- | --- | --- | --- | --- |
| Staphylococcus | 0.77% | 44.91% | 4.07% | 4.12% | 10.25% | 2.50% | 57.62% |
| Carnimonas | 0.00% | 0.00% | 0.00% | 0.01% | 0.02% | 0.00% | 31.71% |
| Brevibacterium | 0.07% | 0.48% | 0.04% | 0.06% | 0.40% | 0.01% | 5.41% |
| Corynebacterium | 7.44% | 2.68% | 40.32% | 1.78% | 2.43% | 41.49% | 3.37% |
| Turicella | 0.00% | 0.00% | 0.01% | 0.24% | 0.00% | 0.04% | 1.12% |
| Acinetobacter | 0.02% | 22.25% | 0.03% | 0.39% | 18.41% | 0.01% | 0.05% |
| Psychrobacter | 0.00% | 16.53% | 0.00% | 0.06% | 0.48% | 0.00% | 0.05% |
| Turicibacter | 0.38% | 0.09% | 0.10% | 0.73% | 0.03% | 0.41% | 0.04% |
| Alloiococcus | 0.00% | 0.00% | 0.07% | 0.30% | 0.00% | 2.16% | 0.03% |
| Auritidibacter | 0.00% | 0.00% | 0.03% | 1.04% | 0.01% | 0.25% | 0.03% |
| Francisella | 1.02% | 2.26% | 0.09% | 72.01% | 0.53% | 0.22% | 0.02% |
| Clostridium XI | 1.29% | 0.06% | 0.29% | 1.04% | 0.10% | 0.33% | 0.02% |
| Fusobacterium | 0.00% | 0.00% | 0.98% | 0.00% | 0.21% | 4.57% | 0.01% |
| Brachybacterium | 0.08% | 0.65% | 0.04% | 0.00% | 0.01% | 0.02% | 0.01% |
| Wautersiella | 0.00% | 1.37% | 0.00% | 0.00% | 0.06% | 0.00% | 0.00% |
| Klebsiella | 0.01% | 0.04% | 0.03% | 0.00% | 8.95% | 0.03% | 0.00% |
| Clostridium sensu stricto | 0.36% | 0.03% | 0.07% | 0.65% | 0.04% | 0.12% | 0.00% |
| Pseudomonas | 0.02% | 1.98% | 0.00% | 0.01% | 14.47% | 0.00% | 0.00% |
| Peptostreptococcus | 0.00% | 0.01% | 1.67% | 0.00% | 0.63% | 1.51% | 0.00% |
| Streptococcus | 0.17% | 0.00% | 15.60% | 0.00% | 0.02% | 0.66% | 0.00% |
| Ignatzschineria | 0.02% | 0.00% | 0.00% | 0.08% | 16.23% | 0.00% | 0.00% |
| Trueperella | 0.10% | 0.00% | 3.21% | 2.72% | 2.99% | 6.75% | 0.00% |
| Arthrobacter | 1.07% | 0.36% | 0.00% | 0.30% | 0.00% | 0.18% | 0.00% |
| Bacillus | 14.57% | 0.24% | 21.12% | 0.20% | 0.00% | 23.64% | 0.00% |
| Nesterenkonia | 0.00% | 0.00% | 0.02% | 0.03% | 0.00% | 2.93% | 0.00% |
| Massilia | 0.00% | 0.00% | 0.04% | 0.03% | 11.01% | 0.00% | 0.00% |
| Enterococcus | 8.16% | 0.10% | 0.58% | 0.00% | 0.59% | 0.00% | 0.00% |
| Peptoniphilus | 0.02% | 0.00% | 2.98% | 0.00% | 0.78% | 1.92% | 0.00% |
| Salmonella | 0.00% | 1.37% | 0.00% | 0.00% | 0.00% | 0.04% | 0.00% |
| Parvimonas | 0.00% | 0.00% | 0.45% | 0.00% | 0.21% | 1.91% | 0.00% |
| Anaerococcus | 0.30% | 0.00% | 0.12% | 0.00% | 0.69% | 1.19% | 0.00% |
| Propionibacterium | 0.00% | 0.00% | 0.03% | 0.00% | 0.00% | 1.07% | 0.00% |
| Facklamia | 0.06% | 0.63% | 0.03% | 0.00% | 0.26% | 0.10% | 0.00% |
| Murdochiella | 0.00% | 0.00% | 3.82% | 0.00% | 0.46% | 0.62% | 0.00% |
| Helcococcus | 0.00% | 0.00% | 0.25% | 0.00% | 1.16% | 0.19% | 0.00% |
| Proteus | 57.92% | 0.00% | 0.01% | 0.00% | 4.58% | 0.01% | 0.00% |
| Sporosarcina | 2.98% | 0.00% | 0.01% | 0.00% | 0.00% | 0.01% | 0.00% |
| Enhydrobacter | 0.00% | 0.00% | 0.09% | 0.00% | 1.21% | 0.00% | 0.00% |
| Ureibacillus | 0.96% | 0.00% | 0.00% | 0.00% | 0.00% | 0.00% | 0.00% |
| Others | 0.12% | 0.62% | 0.03% | 9.03% | 0.11% | 0.03% | 0.17% |
