# Supplementary material for: Microbial composition in Hyalomma anatolicum collected from livestock in the United Arab Emirates using next-generation sequencing
Source: Parasit Vectors. 2022 Jan 20;15:30. doi: 10.1186/s13071-021-05144-z (PMC8772180; doi:10.1186/s13071-021-05144-z)
Supplement: Supplementary file 6 — Additional file 6: Table S6. Correlation matrix showing pairwise Pearson’s r correlations between genera (bottom) and their associated significance (top). [file 13071_2021_5144_MOESM6_ESM.docx]

**Additional file 2: Table S2.** Microbial phyla (presence in %) detected in *H. anatolicum* adult ticks from three emirates in UAE.

| Phylum | C.D | C.S | G.D | G.S | S.A | S.D | S.S |
| --- | --- | --- | --- | --- | --- | --- | --- |
| Proteobacteria | 59.22% | 45.95% | 0.60% | 82.69% | 76.85% | 0.47% | 32.05% |
| Firmicutes | 31.00% | 47.01% | 53.93% | 8.66% | 16.46% | 41.26% | 57.95% |
| Actinobacteria | 9.51% | 5.35% | 44.24% | 6.49% | 6.01% | 53.46% | 9.99% |
| Bacteroidetes | 0.01% | 1.62% | 0.12% | 0.85% | 0.46% | 0.17% | 0.01% |
| Cyanobacteria/Chloroplast | 0.02% | 0.04% | 0.08% | 0.14% | 0.00% | 0.05% | 0.00% |
| Planctomycetes | 0.00% | 0.00% | 0.00% | 0.13% | 0.00% | 0.00% | 0.00% |
| Verrucomicrobia | 0.00% | 0.00% | 0.00% | 0.02% | 0.00% | 0.00% | 0.00% |
| Euryarchaeota | 0.03% | 0.00% | 0.00% | 0.00% | 0.00% | 0.00% | 0.00% |
| Acidobacteria | 0.00% | 0.02% | 0.00% | 0.00% | 0.00% | 0.00% | 0.00% |
| Chloroflexi | 0.13% | 0.00% | 0.03% | 0.00% | 0.00% | 0.01% | 0.00% |
| Deinococcus-Thermus | 0.01% | 0.00% | 0.00% | 0.00% | 0.00% | 0.00% | 0.00% |
| Fusobacteria | 0.00% | 0.00% | 0.98% | 0.00% | 0.21% | 4.57% | 0.01% |
| Candidatus Saccharibacteria | 0.05% | 0.00% | 0.00% | 0.00% | 0.00% | 0.00% | 0.00% |
| Cloacimonetes | 0.00% | 0.00% | 0.01% | 0.00% | 0.00% | 0.00% | 0.00% |
